# The cobalamin-binding domain of cobalamin-dependent radical *S*-adenosylmethionine enzymes: Familiarity in unfamiliar places

**DOI:** 10.1016/j.jinorgbio.2025.113204

**Published:** 2025-12-22

**Authors:** Dante M. Avalos, Catherine L. Drennan

**Affiliations:** a Harvard Graduate Program in Biophysics, Harvard University, Boston, MA, 02115, United States of America; b Howard Hughes Medical Institute, Massachusetts Institute of Technology, Cambridge, MA, 02139, United States of America; c Department of Biology, Massachusetts Institute of Technology, Cambridge, MA, 02139, United States of America; d Department of Chemistry, Massachusetts Institute of Technology, Cambridge, MA, 02139, United States of America

**Keywords:** Bioorganometallic chemistry, Bioinorganic Chemistry, Radical Chemistry, Methylation, Rossmann fold, Carbon-carbon bond formation

## Abstract

Cobalamin (Cbl)-dependent Radical *S*-adenosylmethionine (RS) enzymes are well known for their use of two powerful cofactors to catalyze chemically challenging reactions, such as methylations on unactivated carbons and phosphorus centers, ring contractions, ring formations, and thioether bond formations. Our repertoire of Cbl-dependent RS enzyme structures has grown since the first solved structure of the oxetanocin A biosynthetic enzyme OxsB in 2017, which has provided insight into the structural basis of catalysis. In particular, the Cbl-binding domains of these RS enzymes have been found to have interesting structural variations that seem to correlate with enzymatic function, at least for the small number of enzymes that have been characterized. In this review, we highlight the recent research about the Cbl cofactor in Cbl-dependent RS enzymes. We compare modes of Cbl binding and demonstrate a previously undetected connection between a subgroup of Cbl-dependent RS enzymes and the corrinoid iron-sulfur protein (CFeSP) from the Wood-Ljungdahl pathway of reductive acetogenesis. Additionally, we discuss recent mechanistic findings on Cbl-dependent RS enzymes OxsB and its close homolog AlsB, which have not been recently reviewed. As Cbl-dependent RS enzymes are involved in making antiviral and antibiotic compounds, herbicides, and other molecules of value, understanding and manipulating enzyme activity has implications in both medicine and agriculture.

## 1. Introduction

**Fig. 1.**
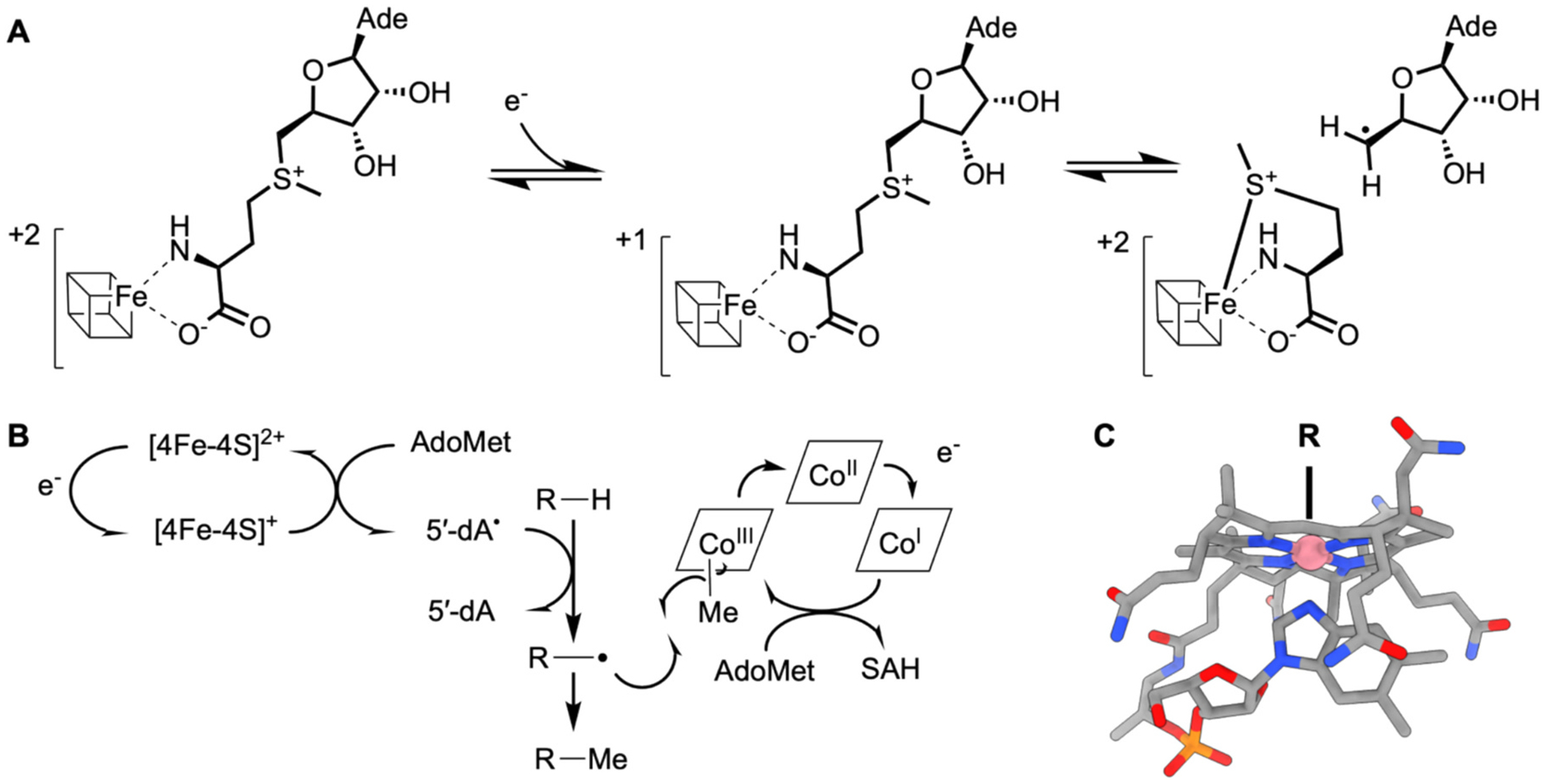
The radical generation and general reaction scheme of Cbl-dependent RS methylases. **A**. The generation of the 5′-dA• species by RS enzymes. **B**. The Cbl-dependent RS enzyme methylase general reaction scheme. **C**. The 3D structure of “Base-on” Cbl. Upper ligand R can be a methyl group (MeCbl) or a 5′-deoxyadenosine moiety (AdoCbl). The lower ligand shown here is 5,6-dimethylbenzimidazole (DMB). Cbls are the subset of corrinoids that contain DMB.

Since being established as an enzyme superfamily in 2001, membership in the Radical *S*-adenosylmethionine (RS) enzyme superfamily has continuously grown, with increasing numbers of RS enzymes having been identified to, and/or predicted to, catalyze chemically difficult reactions [1–8]. RS enzymes are typically characterized by the presence of a partial or full triose-phosphate-isomerase (TIM) barrel with a CX_3_CXϕC motif, where a site-differentiated [4Fe-4S] cluster is coordinated by the three cysteine residues [9,10]. Chelation of the unique iron of the [4Fe-4S] cluster by *S*-adenosylmethionine (AdoMet) allows for reductive cleavage and formation of a 5′-deoxyadenosine radical (5′-dA•) (Fig. 1A) [11,12]. With the notable exception of the cobalamin (Cbl)-dependent RS enzyme TsrM, which does not employ a 5′-dA• intermediate [13], 5′-dA• is typically used by RS enzymes to initiate the enzymatic reaction, usually through a hydrogen atom abstraction (Fig. 1B, left) [14].

**Fig. 2.**
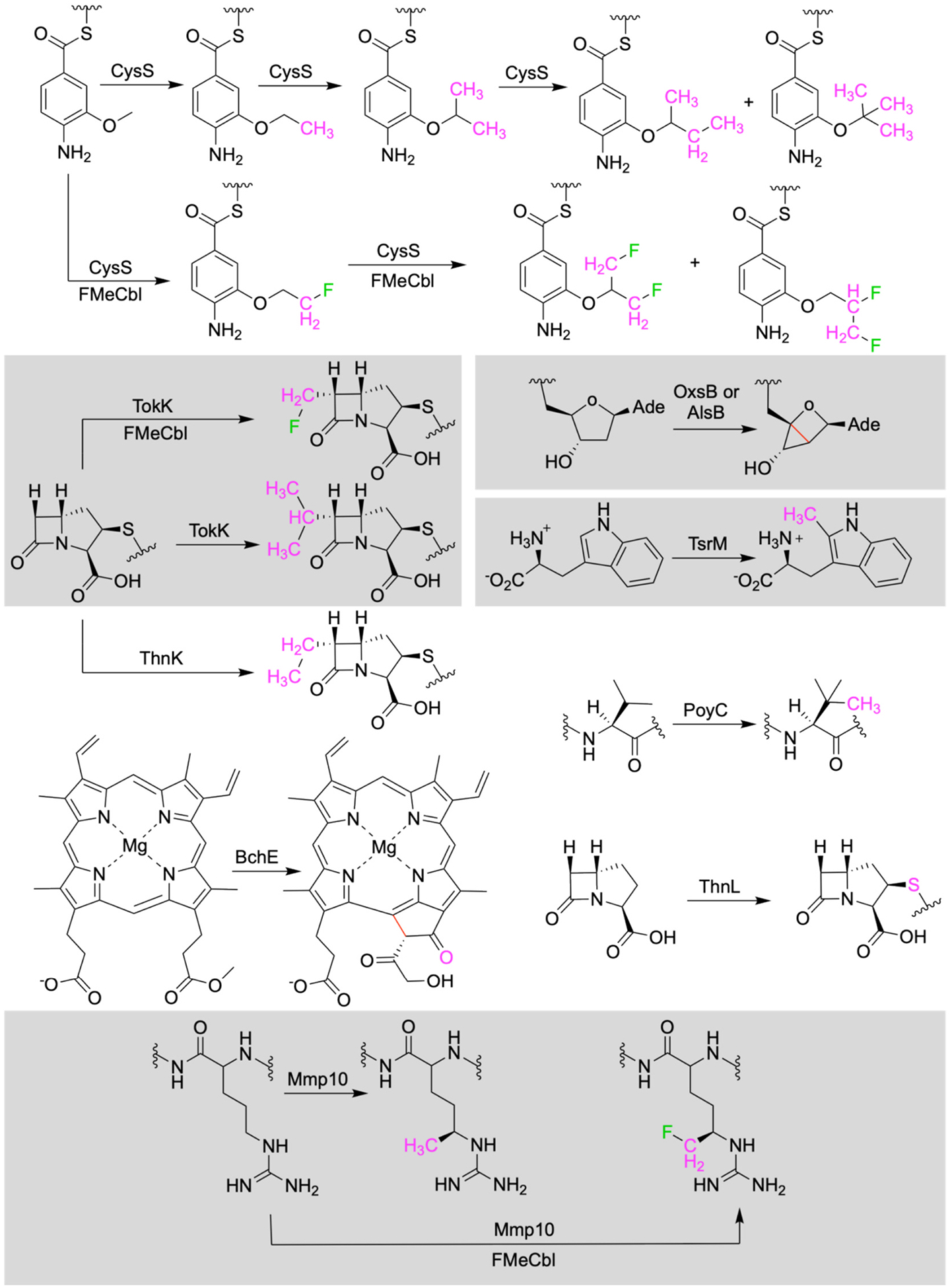
Cbl-dependent RS enzymes catalyze a wide range of chemically challenging reactions. FMeCbl refers to fluoromethylcobalamin. Reactions in grey boxes are catalyzed by structurally characterized enzymes. Bond or atoms added during the reaction are colored magenta. Fluorine atoms that are colored green are added by a fluoromethylation reaction.

**Fig. 3.**
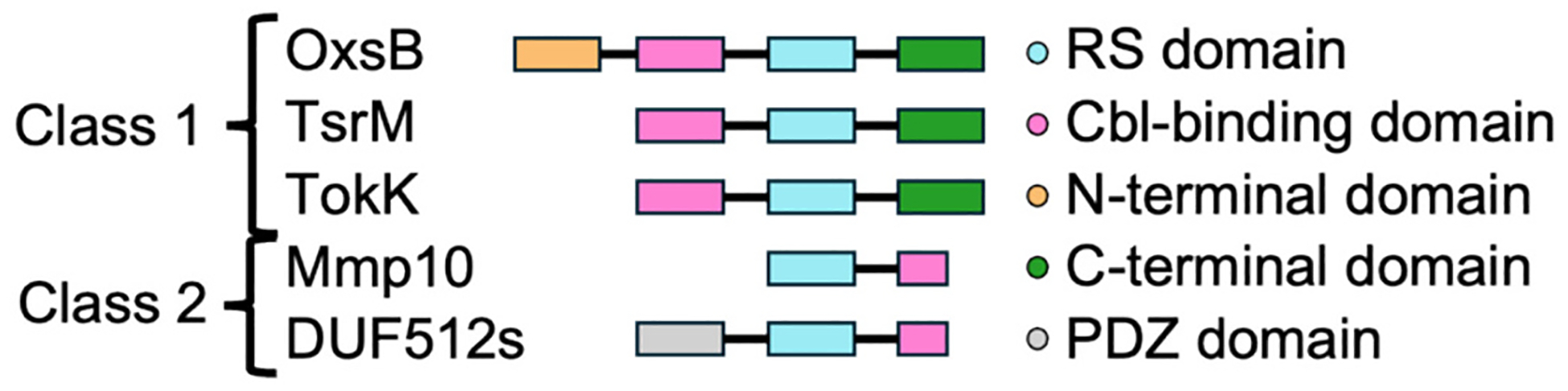
Domain map of all structurally characterized Cbl-dependent RS enzymes. DUF512s refers to DUF512-containing proteins.

Of the RS enzyme superfamily, the Cbl-dependent RS enzymes have remained one of the most fascinating subfamilies because of the riches of cofactors involved in performing their impressive chemistry (Fig. 2). The requirement for Cbl, which is known as nature's most beautiful cofactor, is evolutionarily interesting because Cbl is an expensive cofactor for organisms to synthesize and/or import, especially when compared to the biologically cheaper alternative AdoMet [15–18]. Notably, both AdoMet and methylcobalamin (MeCbl) can perform methyl transfer reactions and both AdoMet and 5′-deoxyadenosylcobalamin (AdoCbl; aka coenzyme B_12_) can generate 5′-dA• and initiate radical chemistry. For this reason, Perry Frey coined AdoMet as the “Poor Man's coenzyme B_12_” [19]. Approximately 30 enzymes are required to biosynthesize Cbl [20], which contains a Co coordinated by four nitrogen atoms of a highly decorated ring structure referred to as the “corrin ring” (Fig. 1C). The most substantial decoration is a “tail” that includes a 5,6-dimethylbenzimidazole (DMB) base. Although all Cbls have a DMB base, the DMB is not conserved among the broader family of corrinoid cofactors for which Cbls are a subgroup. Research on Cbl-dependent RS enzymes has been filled with an abundance of experimental hurdles, largely due to the difficulty of incorporating the appropriate cofactors during heterologous expression in conjunction with the cofactor's oxygen and light sensitivity [21]. Clever methods have been developed by Squire Booker's lab, and others, specifically for studying Cbl-dependent RS enzymes, which has led to a growing number of characterized enzymes [22–24]. Based on sequence similarity networks, there is more than one subgroup of Cbl-dependent RS enzymes [25]. Differences among Cbl-dependent RS enzymes include the order of the domains, i.e. whether the Cbl-binding domain is N-terminal or C-terminal to the RS domain; the number and types of domains, and size of the Cbl-binding domain (Fig. 3) [26,27]. Here we group Cbl-dependent RS enzymes into two classes based on the domain order (Fig. 3), but we suspect additional groupings will be necessary as we learn more about this enzyme family.

In terms of functional groupings, Cbl-dependent RS enzymes are generally thought of as methyltransferases and non-methyltransferases. These enzymes are especially well known for their ability to do challenging methylations such as methyl transfers onto unactivated *sp*^*2*^- and *sp*^*3*^-hybridized carbon centers utilizing MeCbl and AdoMet together (Fig. 2) [14,28]. Cbl-dependent RS methyl transfer enzymes, such as PoyC and CysS, can catalyze either single or multiple methyl transfers onto a substrate [29,30]. In the non-methyltransferase category, Cbl-dependent RS enzymes have been reported to catalyze ring contractions (OxsB and AlsB), thioether bond formation (ThnL), and ring formation (BchE) reactions (Fig. 2) [31–34]. For these non-methyltransferase Cbl-dependent RS enzymes, the role of the Cbl and the required oxidation state of the Cbl is often not clear. For OxsB, Cbl is known to be required for enzyme activity, but the role of the cofactor and the oxidation state required for catalysis have been enigmatic (discussed further below) [31,35]. Recently, efforts to engineer Cbl-dependent RS enzymes as biocatalysts have been explored by the laboratories of Squire Booker and Min Dong, demonstrating the ability of Cbl-dependent RS enzymes to fluoromethylate substrates by incorporating fluoromethylcobalamin [36,37] (Fig. 2).

Similar to general RS enzymes, methyl transfer by Cbl-dependent RS enzymes involves a hydrogen atom abstraction on the substrate by a 5′-dA• derived from AdoMet (Fig. 1A,B) [28]. AdoMet is also used to generate MeCbl by a nucleophilic attack from cob(I)alamin, followed by, what is generally assumed to be, a homolytic methyl transfer to the substrate, forming cob(II)alamin and a methylated product (Fig. 1B). Cob(I)alamin is regenerated from cob(II)alamin through a single electron reduction. As a result of catalysis, two cleavage products of AdoMet can be detected: 5′-deoxyadenosine (5′-dA), generated from reductive cleavage of AdoMet, and *S*-adenosylhomocysteine (SAH), formed from AdoMet methyl transfer to Cbl. Though TsrM uses AdoMet to form MeCbl, as mentioned above, it does not reductively cleave AdoMet, and thus no 5′-dA was detected for this Cbl-dependent RS enzyme [38]. The structure, function, and mechanism of TsrM, and how it compares to other Cbl-dependent RS enzyme methylases was covered elegantly in a separate review [14]. There is also an excellent detailed review by Susan C. Wang that covers mechanistic studies of Cbl-dependent RS enzymes [39]. To avoid duplication of content, we will not delve into the mechanism of methyl transfer by Cbl-dependent RS enzymes in this focused review.

## 2. The majority of the non-RS Cbl-dependent enzymes bind Cbl “base-off His-on”

**Fig. 4.**
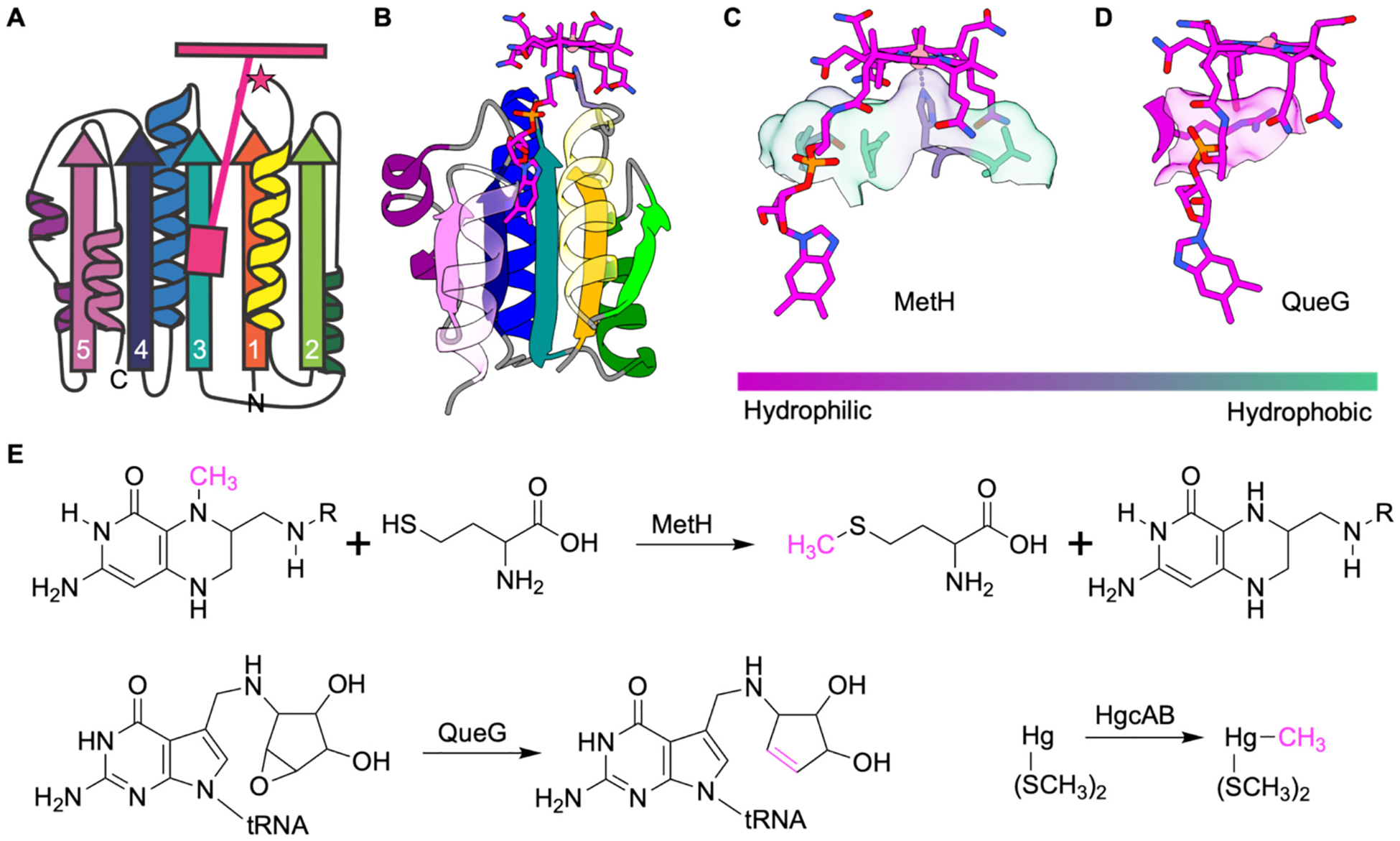
Cbl-dependent enzyme methionine synthase uses a 5-stranded Rossmann fold and a histidine residue for “Base-off His-on” Cbl binding. **A**. A topological depiction of the classic Cbl-binding Rossmann domain of MetH (PDB ID: 1BMT) [41]. Histidine ligand to Cbl (magenta) is indicated with a magenta star. Strands are numbered according to position in the sequence. **B**. Ribbon drawing of the Cbl-binding domain of MetH colored to match the topology diagram. Cbl and His are drawn in sticks. **C**. Cbl in MetH (PDB ID: 1BMT) [41] is coordinated (dashed lines) by a His residue that is packed against a Leu side chain. A hydrophobicity scale [65] shows a hydrophobic environment (green surface) below the corrin ring except for the His ligand. **D**. Cbl in QueG (PDB ID: 5D0A) [49] is not coordinated by a lower ligand. An Arg side chain (sticks) blocks water coordination in a hydrophilic environment (purple surface). **E**. Schemes of the reactions catalyzed by MetH, QueG, and HgcAB.

Prior to the first crystal structure of protein-bound Cbl, theories existed as to how the lower ligand of free Cbl, the DMB, might be modulating the properties of the Cbl to either stabilize or destabilize the biologically relevant forms of the Cbl cofactors: MeCbl and AdoCbl (Fig. 1C) [40]. Thus, it came as a surprise when the first structure of a protein-bound Cbl in 1994 showed that a protein residue, a histidine, had replaced the DMB as the lower ligand (Fig. 4A–C) [41]. That structure, which was of the Cbl-binding domain of the methyltransferase methionine synthase (MetH) (Fig. 4A–C,E), revealed that Cbl-dependent enzymes utilize a common nucleotide-binding fold, the Rossmann fold, which is typically comprised of 5–6 parallel β-strands sandwiched by α-helices (Fig. 4A,B). Though the parallel β-strands are adjacent to each other structurally, the primary sequence alternates between β-strands and α-helices. The first strand (based on sequence) is in the center of the sheet, the second strand of a five-stranded sheet is on the outside edge of the sheet, whereas the third strand is next to the first strand in the center of the sheet with the fourth and fifth strands on the opposite edge of the sheet from strand 2 (Fig. 4A,B). This arrangement of strands creates a cavity between the cross-over strands numbered 1 and 3 in the case of MetH (Fig. 4A,B), into which the nucleotide binds. The corrin ring of Cbl sits in this position between strands 1 and 3. A histidine residue from the loop that follows strand 1, referred to as the “His loop”, coordinates the Co of the Cbl, and a leucine residue from the loop that follows strand 3 stacks against the histidine side chain. The DMB-base of Cbl penetrates the domain residing between the β-sheet and α-helices. This Cbl-binding mode is termed the “Base-off His-on” Cbl binding mode (Fig. 4B,C). The Base-off Cbl binding mode allows for the local protein environment to modulate the reactivity of Co [41,42]. For example, a smaller histidine side chain is easier to reposition than the bulky DMB so that the lower ligand to Cbl can move closer to Co to stabilize the 6-coordinate MeCob (III)alamin state and move farther to stabilize the 4-coordinate cob(I) alamin state that is formed after methyl transfer in MetH [41,43]. The properties of the histidine side chain can also be tuned through hydrogen bonding, yielding a histidine side chain with more imidazolate-like character that is a stronger ligand to cob(III)alamin or generating a neutral or positively charged histidine that does not coordinate the cob(I)alamin species. Tuning the properties of the DMB is not possible. Therefore, although the substitution of the DMB with histidine was a surprise, in retrospect, this substitution makes chemical sense. Later work showed that histidine can be a ligand to Cbl for AdoCbl-dependent enzymes in addition to the MeCbl-dependent MetH [44,45]. Though a His-ligand is typical for Cbl-dependent enzymes as is the use of a Rossmann fold, there are exceptions. For example, Cbl-dependent mercury methylase HgcA is thought to have a cysteine residue as the lower axial ligand [46–48], and Cbl-dependent queuosine biosynthetic enzyme QueG has been shown through crystallography to use an arginine side chain to block access to the lower face of the corrin ring, preventing lower axial Co coordination and thereby stabilizing a 4-coordinate cob(I)alamin species [41], which can be used as for nucleophilic attack on the epoxyqueuosine substrate (Fig. 4D,E) [49].

## 3. Many Cbl-dependent RS enzymes bind Cbl “Base-off” but not “His-on” using a Rossmann fold

**Fig. 5.**
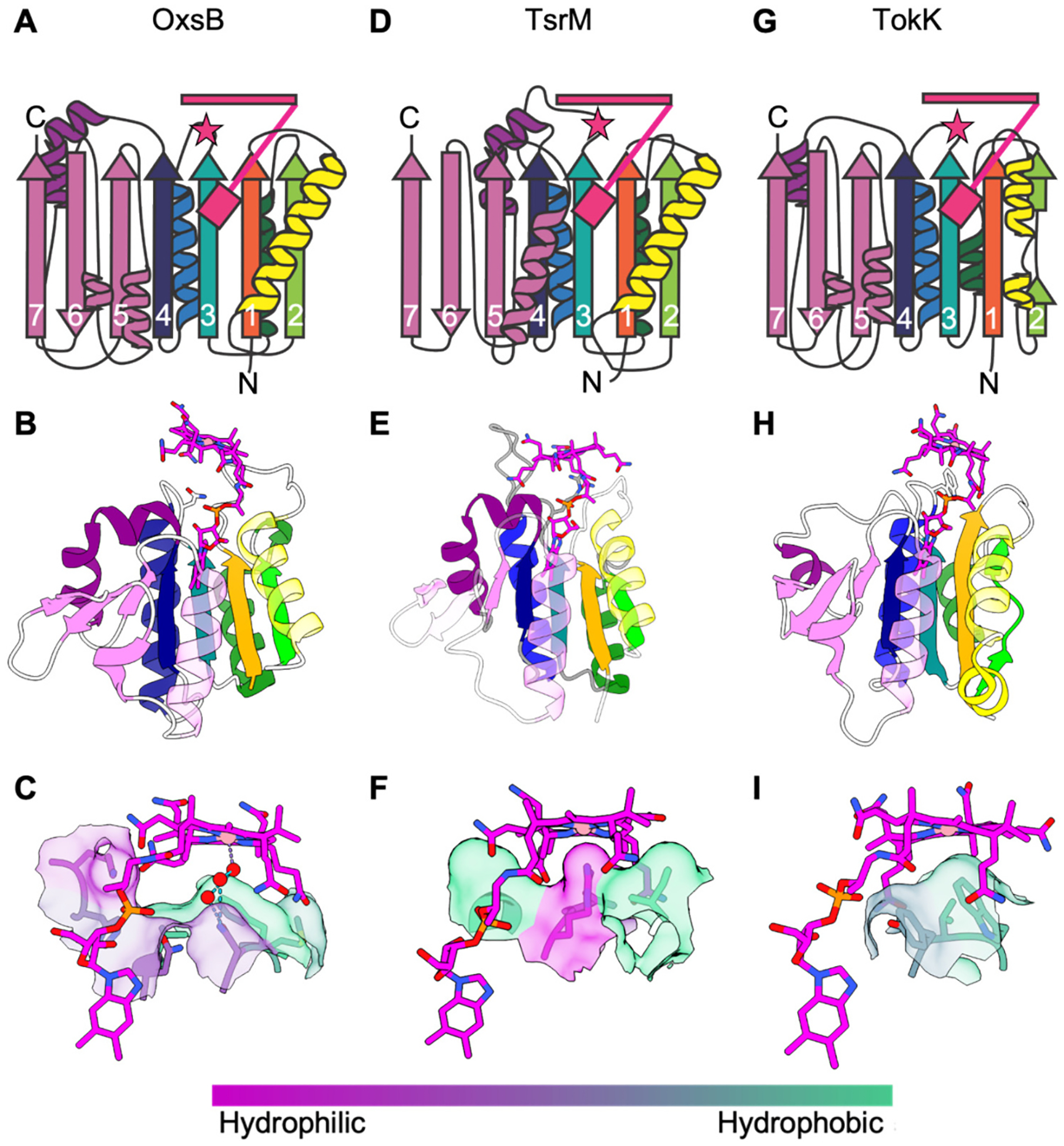
The Cbl-binding domains of class 1 Cbl-dependent RS enzymes utilize a 5-stranded Rossmann fold with a C-terminal β-hairpin. **A**. Topology diagram of OxsB (PDB ID: 5UL4) [31]. **B**. Ribbon drawing of OxsB (PDB ID: 5UL4) [31]. **C**. The region below the Co in OxsB (PDB ID: 5UL4) [31] colored by hydrophobicity [65]. **D**. Topology diagram of TsrM (PDB ID: 6WTF) [51]. **E**. Ribbon drawing of TsrM (PDB ID: 6WTF) [51]. **F**. The region below the Co in TsrM (PDB ID: 6WTF) [51] colored by hydrophobicity [65]. **G**. Topology diagram of TokK (PDB ID: 7KDY) [50]. **H**. Ribbon drawing of TokK (PDB ID: 7KDY) [50]. **I**. The region below the Co in TokK (PDB ID: 7KDY) [50] colored by hydrophobicity [65]. Amino acid residues closest to the lower axial position of the Co ion are indicated with a magenta star in panels A, D, and G. Secondary structures in panels are colored in sequence order. Cobalamin is shown in magenta in panels B-I. Secondary structures after and including β5 are violet.

As of this review, four X-ray crystal structures of full sequence Cbl-dependent RS enzymes have been solved, the non-methyltransferase OxsB, and the methyltransferases TokK, TsrM, and Mmp10 [26,31,50,51] (reactions shown in Fig. 2); and one structure of just the Cbl-binding domain of a Cbl-dependent RS enzyme, TM0182 (N-TM0182) [52]. Unfortunately, the latter structure lacks bound Cbl, prohibiting structural comparisons. Therefore, this review will focus on the four full sequence structures, some of which were solved with substrates bound (Table 1). OxsB was the first structure of the Cbl-dependent RS enzymes to be solved [31]. The structure revealed a domain architecture that included four domains: a Cbl-binding domain, a RS enzyme domain, a C-terminal helical bundle domain, and a N-terminal domain (Fig. 3). The Cbl-binding domain of OxsB exhibited a Rossmann fold that resembled MetH (r.m.s.d. of 3.10 Å) [31], with the exception that OxsB has an additional β-hairpin following strand 5 of the Rossmann fold (Fig. 5A,B). Structures of TokK and TsrM showed similar domain architecture to OxsB, though both TokK and TsrM lack the N-terminal domain of unknown function (Fig. 3) [31,50,51]. The Cbl-binding domains of TokK and TsrM closely resemble the Cbl-binding domain of OxsB with the 5-stranded Rossmann fold and C-terminal β-hairpin (Fig. 5D,E,G,H). Strand 2 in TokK is split into 2 smaller β-strands, though this does not cause any consequential changes to the binding of Cbl as strand 2 does not make contact with Cbl (Fig. 5G,H). For the remainder of this review, we define OxsB, TsrM, and TokK as class 1 Cbl-dependent RS enzymes due to their structural similarity (Fig. 3).

All four of the structurally characterized Cbl-dependent RS enzymes uniquely capitalize on the ability to modulate the Co reactivity with the local protein environment. OxsB binds Cbl ‘Base-off’ but instead of using a histidine ligand, it uses a network of waters to coordinate the Co via Asn186, which is from the loop that follows strand 3 (Fig. 5A–C) [31]. Asn186 occupies the position of the Leu in MetH (Fig. 4C). In TsrM, Arg69, also located in the loop following strand 3, blocks access to the lower face of Cbl (Fig. 5F) without acting as a lower ligand itself [13]. As mentioned above, TsrM does not use the standard proposed mechanism for Cbl-dependent RS that is shown in Fig. 1B, instead transferring the methyl group from MeCbl to substrate by heterolytic cleavage of the Co-C bond, forming a cob(I)alamin species [51]. Cob(I)alamin, as noted above, would be stabilized by the lack of a lower ligand due to the repulsion between a ligand and the Co(I) ${\text{d}}_{\text{z}}^{2}$ orbital. The presence of a positively charged Arg69 would additionally stabilize the highly reduced cob(I)alamin [51]. TokK also uses a residue in the loop following strand 3 to occupy the space under the Co, but instead of a His or Arg or Asn residue, TokK employs Trp76, a large, hydrophobic amino acid that cannot act as a lower ligand (Fig. 5I) [50]. Spectroscopic data supports the finding of a 4-coordinate cobalt metal center in TokK [50]. Given that TokK is thought to proceed by a radical-based methyl transfer mechanism that generates cob(II)alamin (Fig. 1B), which is a form of Cbl that prefers 5-coordinate geometry, it is interesting that the Cbl in TokK lacks a lower ligand. Although a 4-coordinate Cbl should favor cob(I) alamin, the hydrophobic nature of Trp should run counter to that preference. Consistent with the latter, substitution of Trp to Lys has been shown to lower TokK activity [50]. Additionally, sequence alignment suggests CysS and ThnK likely also place a Trp residue underneath the Co of the Cbl [53]. Summaries and comparisons of other aspects of TsrM and TokK structures have been covered in several reviews [14,16,28].

## 4. A modified Cbl-binding domain in the modified position with respect to the RS domain was observed in the structure of Cbl-dependent RS enzyme Mmp10

**Fig. 6.**
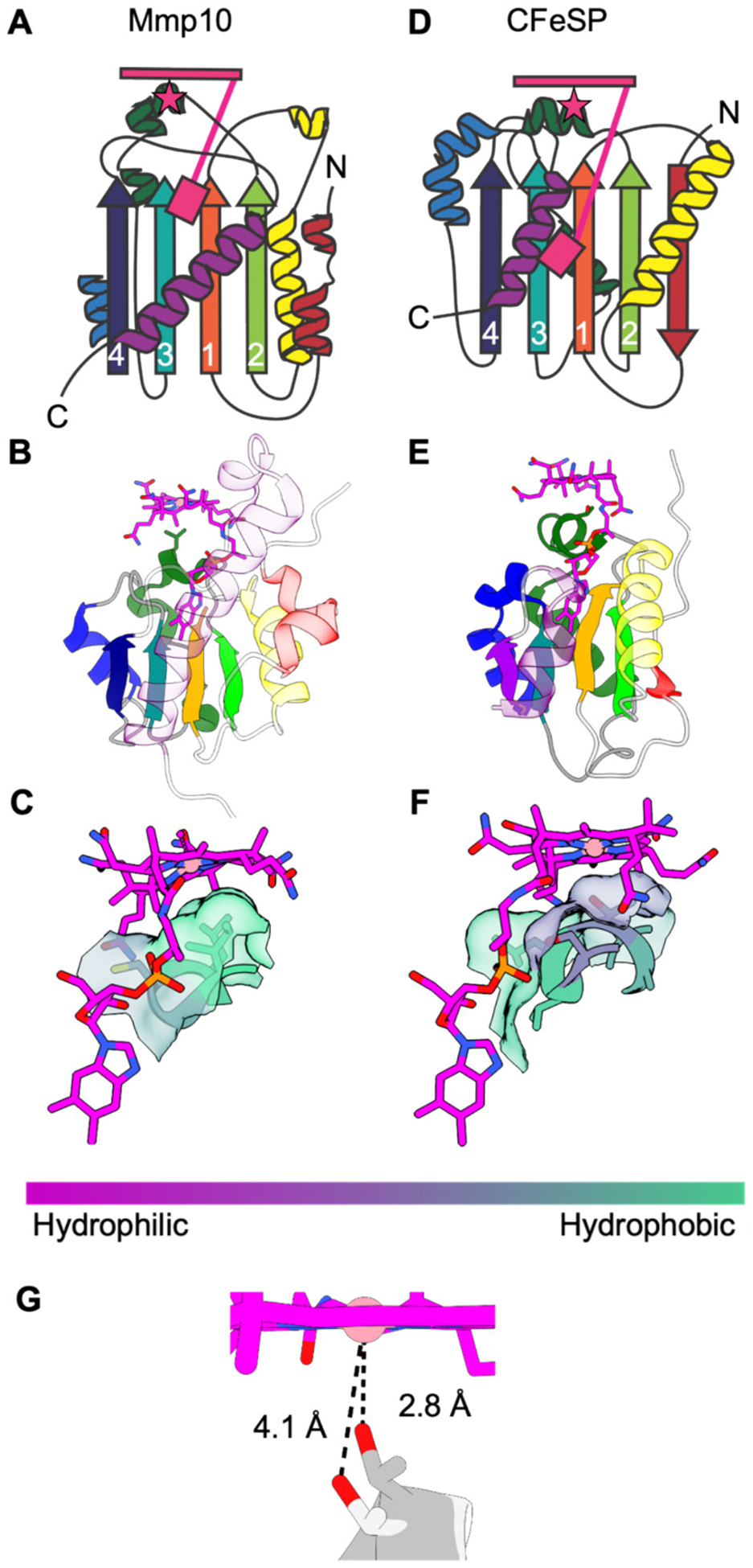
The Cbl-binding domains of the class 2 Cbl-dependent RS enzyme Mmp10 and CFeSP are similar. **A**. Topology diagram of Mmp10 (PDB ID: 7QBS) [26]. **B**. Ribbon depiction of Mmp10 (PDB ID: 7QBS) [26]. **C**. The region below the Co in Mmp10 (PDB ID: 7QBS) [26] colored by hydrophobicity [65]. **D**. Topology diagram of CFeSP (PDB ID: 4DJD) [57]. **E**. Ribbon depiction of CFeSP (PDB ID: 4DJD) [57]. F. The region below the Co in CFeSP (PDB ID: 4DJD) [57] colored by hydrophobicity [65]. Amino acid residues closest to the lower axial position of the cobalt ion are indicated with a magenta star in panels A and D. Secondary structures in panels are colored in sequence order. Cobalamin is shown in magenta in panels B, C, E, and **F. G**. The distances from the carboxyl group of Thr378 from *Moorella thermoacetica* CFeSP (light grey, PDB ID: 4DJE) [57] and Thr375 from *Clostridium autoethanogenum* (dark grey, PDB ID: 9FZZ) [62].

Of the four structures of Cbl-dependent RS enzymes, Mmp10 is regarded as the major structural outlier (Fig. 6A,B) [26,28]. Mmp10 is the only characterized Cbl-dependent RS enzyme where the Cbl-binding domain is C-terminal rather than N-terminal to the RS domain (Fig. 3) [26]. Additionally, whereas the other Cbl-dependent RS enzymes share a nearly identical Cbl-binding domain with 5 parallel β-strands forming a β-sheet sandwiched by helices (i.e. a Rossmann fold), Mmp10 uses a Cbl-binding domain containing only 4 parallel β-strands surrounded by helices (Fig. 6A,B) [26]. Furthermore, a short helix from strand 2 sits below the corrin ring, which is not found in the other Cbl-dependent RS enzymes (Fig. 6A,B) [26].

## 5. Mmp10 and DUF512-containing enzymes appear to be part of a new structural class of Cbl-dependent RS enzyme

With the Mmp10 structure showing an unusual 4-stranded Cbl-binding domain in the atypical position of being C-terminal to the RS domain. Wang et al. (2024) investigated the possibility of more Cbl-dependent RS enzymes existing with comparable Cbl-binding domains positioned similarly [27]. They found candidates with a domain of unknown function (DUF), annotated DUF512, following predicted RS domains (Fig. 3). One of these DUF512-containing RS enzymes also contained a PSD-95, DLG and ZO-1 (PDZ) domain at the N-terminus (Fig. 3). Though PDZ domains usually govern protein-protein interactions, the role of the PDZ domain in DUF512-containing proteins is unknown, and no other RS enzymes appear to have PDZ domains [54]. Wang et al. (2024) isolated four DUF512-containing proteins and solved the X-ray crystal structures of two of those proteins (Table 1) [27]. Both structures showed Cbl-binding domains consisting of a 4-stranded Rossmann fold, reminiscent of Mmp10 described above. Additionally, as observed for Mmp10, a short helix that follows strand 2 runs under the corrin ring [27]. Although the roles of these DUF512-containing proteins remain unknown, biochemical analysis demonstrated that all four proteins can convert hydroxocobalamin (OHCbl) to MeCbl with the addition of AdoMet, producing SAH [27]. Furthermore, all four DUF512-containing proteins convert AdoMet into 5′-dA and methionine, consistent with radical methyltransferase activity [27]. Though the DUF512-containing proteins are not currently annotated as Cbl-dependent RS enzymes, this preliminary work suggests that they are, in fact, Cbl-dependent RS enzymes. When the DUF512-containing protein sequences are considered, the Cbl-dependent RS enzyme family will grow by an additional ~6000 sequences. We refer to Mmp10 and DUF512-containing proteins as class 2 Cbl-dependent RS enzymes due to their shared Cbl-binding domains and general domain architecture (Fig. 3).

## 6. The second class of Cbl-dependent RS enzymes bind Cbl “Base-off” but not “His-on” using a Cbl-binding fold first observed in the ancient pathway of reductive acetogenesis

Although the Cbl-binding domain of Mmp10 and DUF512 is unique within the family of Cbl-dependent RS enzymes, it bares striking resemblance to the Cbl-binding domain of the corrinoid iron-sulfur protein (CFeSP) (Fig. 6) of the Wood-Ljungdahl pathway of reductive acetogenesis [55–57]. As far as we know, this structural similarity has not been previously reported. The Wood-Ljungdahl pathway allows acetogens to convert two molecules of carbon dioxide gas into the two carbon units of acetyl-CoA using reducing equivalents from H_2_ gas. Although the Wood-Ljungdahl pathway undoubtedly has ancient origins, this pathway is active in modern acetogenic bacteria, producing an estimated 10^9^ tons of acetate from carbon dioxide each year [58]. In this pathway, the corrinoid cofactor of CFeSP accepts a methyl group from methyltetrahydrofolate and delivers it to a nickel ion in the A-cluster of acetyl-CoA synthase (ACS) where it is incorporated into acetyl-CoA [59]. Notably, both MetH and CFeSP utilize a cob(I)alamin super-nucleophile to remove a methyl group from methyltetrahydrofolate, and both utilize methylcob(III)alamin to methylate their second substrate, transferring the methyl moiety as “${\text{CH}}_{3}^{+}$” and regenerating cob(I)alamin [60,61]. Thus, it is interesting that the Cbl-binding domain architecture differs between MetH and CFeSP as these enzymes are functionally very similar. Prior to the structural analyses of CFeSP [55–57,62], it was thought that the fold variation could be due to the difference in the identity of the base between Cbl, DMB, and the corrinoid of CFeSP, 5-methoxybenzimidazole (OMe-DMB), but the CFeSP structure showed that both bases can be easily accommodated, which perhaps is unsurprising given the similarity. However, some corrinoids have substantially different appendices (an adenine base or *p*-cresol group in place of the DMB for example) [63,64], and CFeSP's corrinoid-binding domain may have evolved to bind an ancestral corrinoid.

As described above for Mmp10, the Cbl-binding domain of CFeSP has 4 parallel strands forming a β-sheet (Fig. 6D,E). Strand 1 is in the center of the sheet with strand 2 on the edge of the β-sheet. Strand 3 is again in the center next to strand 1, with strand 4 on the other β-sheet edge. Helices follow each strand and sandwich the β-sheet. In other words, this smaller Cbl-binding domain follows the same arrangement of strands and helices of all Rossmann folds. There are also structural elements outside of the 4-stranded Rossmann domain; CFeSP has an antiparallel β-strand preceding strand 1 whereas Mmp10 and DUF512 have an α-helix (Fig. 6A,B,D,E). As with all other structures of Cbl-binding domains, the DMB tail sits in-between the sheet and helices, past the strand cross-over point, and the corrin ring sits at the cross-over point above strands 1 and 3 (Fig. 6A,B,D,E). A major difference between the Cbl-binding domains of CFeSP and Mmp10, compared to that of MetH and class 1 Cbl-dependent RS enzymes TsrM, TokK, OxsB, is a short “cap helix” that runs under the corrin in CFeSP and Mmp10 (Fig. 6A,B,D,E). As noted above, residues from loops of the cross-over strands 1 and 3 (His in MetH, Arg in TsrM, Trp in TokK, Asn-water in OxsB) fill the cavity at the cross-over point that is under the corrin, either acting as a lower ligand or blocking a lower ligand. In contrast for CFeSP and Mmp10, a short helix off of strand 2 fills in the cavity underneath the corrin (Fig. 6A,B,D,E). Although side chains from the helix do sit under the corrin and one could potentially ligate the Co, the positions that side chains from a helix can adopt is far more limited than the positions that side chains from a loop can adopt. Thus, the presence of a helix under the corrin makes the CFeSP/Mmp10 Cbl-binding fold less adaptable for modulating Cbl reactivity through side chain positioning. The closest side chain to the Co in CFeSP is a Thr. The distance between the Thr and Co varies in the different structures from 2.8 to 4.1 Å (Fig. 6F–G) [55–57,62]. It should be noted that the Cbl-binding domain of CFeSP is highly flexible resulting in high B-factors and poor-quality density. In most of the maps, density for the Thr side chain is not well defined. In the map with the best density for Thr, the distance for the Thr hydroxyl group to Co is 3.2 Å, too long to be considered a coordination, but too close to allow a water molecule to coordinate. Additional spectroscopic studies should be able to resolve whether Thr is a lower ligand to Co in any, or all, of the Cbl oxidation states. Currently, we can say that CFeSP differs from MetH in that it doesn't have a lower ligand like His whose charged state can be tuned to favor a His-on methylcob(III)alamin state (Fig. 4C) or a His-off cob(I)alamin state.

Instead of a Thr contributed by the cap helix, the closest residue to Co in Mmp10 and in DUF512 is a Leu residue (Fig. 6C) [26] [27]. The presence of Leu322 in Mmp10 appears to block water ligation to Co, yielding the most hydrophobic environment below the corrin ring of any of the structurally characterized Cbl-dependent RS enzymes, based on the Eisenberg hydrophobicity scale (Fig. 6C) [65]. The second most hydrophobic is TokK (Fig. 5I), which, like Mmp10, is proposed to transfer the methyl group as a methyl radical (Fig. 1B). In terms of providing a hydrophobic environment, both variations of the Cbl-binding fold would seem to work well, raising the question of why TokK uses the MetH-like Rossmann fold whereas Mmp10 uses the CFeSP-like Rossmann fold. As more DUF512-containing proteins are confirmed as class 2 Cbl-dependent RS enzymes, we can start to consider whether the type of organism predicts the fold usage or if organism lifestyle, aerobic vs anaerobic for example, provides predictive power. Prior to the Mmp10 structure, the CFeSP-like Cbl-binding Rossmann fold had not been known to exist in non-CFeSP enzymes. Unfortunately, authors of the Mmp10 structure paper didn't recognize the Cbl-binding fold and stated that Mmp10 used a novel binding mode for Cbl [26]. Instead, it is undoubtedly the oldest fold for Cbl binding [66]. However, its use in Cbl-dependent RS enzymes was unexpected and warrants further analysis.

## 7. Role of Cbl in OxsB and AlsB is starting to come into focus

The irony did not escape the authors of the OxsB structural study that this first structure of a Cbl-dependent RS enzyme was of a non-methyltransferase for which the function of Cbl was not known [31]. Excitingly, advances have been made in the biochemical characterization of OxsB and its homolog AlsB that provide insight into the Cbl's role in these non-methyltransferase Cbl-dependent RS enzymes. These advances warrant comment, causing us to expand this otherwise structure-based review article to describe what is now known about OxsB and AlsB reactions in general, and the role of Cbl in particular.

**Fig. 7.**
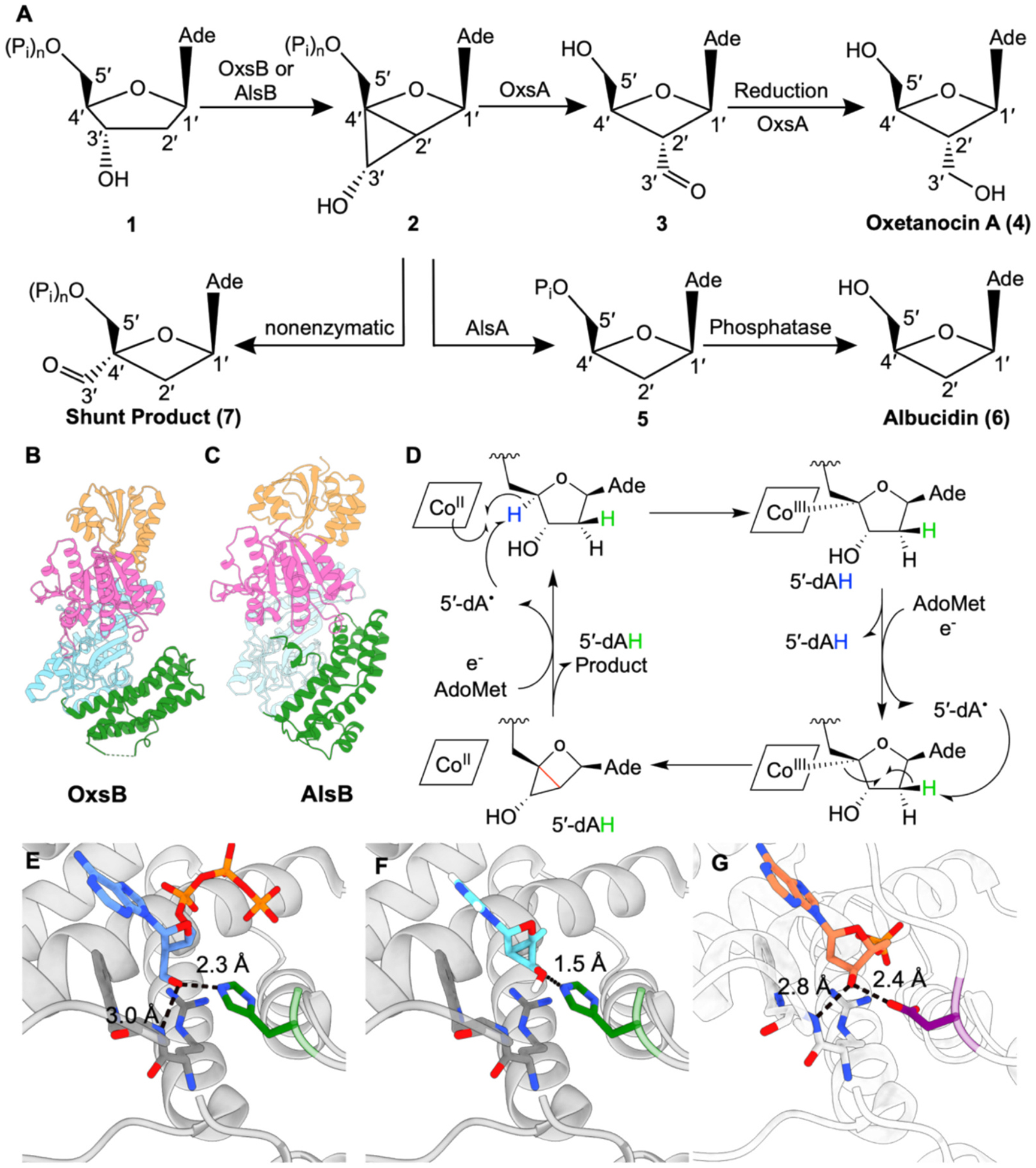
The biosynthetic pathways of OXT-A and Albucidin share similar Cbl-dependent RS enzymes catalyzing a ring contraction. **A**. The biosynthetic pathways of OXT-A and Albucidin. The phosphatase is a nonspecific phosphatase in the cell. Calf Intestinal Phosphatase is the standard in experiments. *n* = 1 for AlsB or 3 for OxsB. **B**. The crystal structure of OxsB containing Cbl, a [4Fe-4S] cluster, and AdoMet (PDB ID: 5UL4) [31]. **C**. The predicted structure of AlsB by AlphaFold 3 [72,73]. B and C are colored by the four domains: the N-terminal domain (orange), the Cbl-binding domain (pink), the RS domain (light blue), and the C-terminal domain (green). **D**. A proposed mechanism for OxsB and AlsB catalysis, including a substrate-cob(III)alamin adduct intermediate. **E**. The distance of His75 (green) in OxsA (grey) (PDB ID: 5TK7) [68] accommodates the smaller OXT-A triphosphate substrate (blue). **F**. The [2.1.0]-fused bicyclic compound (cyan) is too large to fit into the active site of OxsA (grey) (PDB ID: 5TK7) [68]. **G**. The smaller Asp77 (purple) is used instead of a His residue to accommodate the larger 2′-dAMP substrate (orange) in YfbR (light grey) (PDB ID: 2PAU) [75]. Hydrogen bonds are indicated by black dashed lines with their distances located adjacently in panels *E*-G.

OxsB is involved in the biosynthesis of the antiviral, antitumor, and antibacterial oxetane-containing nucleoside analog Oxetanocin-A (OXT-A) (Fig. 7A, 4), catalyzing a ring contraction of the furanose in deoxyadenosine-5′-monophosphate (2′-dAMP), deoxyadenosine-5′-diphosphate (2′-dADP), and deoxyadenosine-5′-triphosphate (2′-dATP) (**1**) with a preference for 2′-dATP (Fig. 7A) [67]. The biosynthetic pathway of **4** contains another enzyme: the His-Asp (HD) domain phosphohydrolase OxsA [68], which was shown to hydrolyze one, two, or three phosphate groups from **4**, rendering this chemical warfare agent inactive for transport out of the organism that biosynthesizes it [68]. Furthermore, it has been reported that a nonspecific alcohol dehydrogenase from lysate is required for aldehyde reduction en route to **4** production, and that OxsB requires the presence of OxsA to prevent shunt product formation (**7**) (Fig. 7A) [31,35,69]. Although direct binding of OxsA to OxsB has not been reported, when OxsA and OxsB are separated by a semi-permeable membrane, no OxsB activity is observed [35].

AlsB, a Cbl-dependent RS enzyme similar to OxsB (44.1 % sequence similarity), was discovered in the biosynthetic pathway of the herbicide Albucidin (6) [32,70]. As expected from the sequence similarity, the predicted structure of AlsB by AlphaFold is highly similarly to OxsB, consisting of the same four domains (Fig. 7B,C) [71–73]. Together, **6** and **4** comprise the only two known naturally occurring oxetane-containing nucleoside analogs [69,74]. Production of **6** (Fig. 7A) differs from that of **4** in two important ways. First, there is no specialized phosphohydrolase like OxsA required for AlsB activity. Instead, production of **6** requires a second RS enzyme; AlsA [32]. Second, AlsB is specific for 2′-dAMP and cannot turnover 2′-dATP, OxsB's preferred substrate [32]. Despite much effort, the product of AlsB and the substrate for AlsA have been enigmatic.

Recently, the laboratory of Hung-wen Liu discovered that AlsB makes a highly unstable [2.1.0]-fused bicyclic compound (**2**), and further established that OxsB is also capable of making this same compound (Fig. 7A) [67]. AlsB and OxsB were additionally shown to be interchangeable in these pathways if the proper substrate for each was used (2′-dAMP or 2′-dATP). The characterization of **2** finally links together the functionality of OxsB and AlsB while providing a possible explanation for the spatial dependence for OxsA by OxsB. In this recent manuscript, authors offer the proposal that OxsA could selectively break the C-C bond between C3′ and C4′ of **2** while removing phosphate groups to form **3**, preventing the nonenzymatic C-C bond breakage between C3′ and C2′ [67]. However, binding of **2** to OxsA would seem to be inconsistent with the structural analyses of OxsA, which revealed modifications to the classic HD domain phosphohydrolase active site to accommodate a smaller four-membered oxetane moiety over a standard five-membered ribose ring (Fig. 7E,G) [68]. If **2** were the true substrate of OxsA, OxsA should have evolved to bind a compound with a larger, not smaller, ring structure, and that is not the case (Fig. 7E,F) [68,75]. Therefore, we postulate here that OxsA binds and dephosphorylates a phosphorylated **3**, or a phosphorylated **4** as originally proposed [68], and that the physical presence of OxsA, through some unknown interaction with OxsB, allows OxsB to hold onto **2** long enough for the correct C3′ and C4′ bond to be broken before product release (Fig. 7A). In contrast, AlsA may act directly on **2** to form **5**. A substrate-bound structure of AlsA would be a valuable confirmation of this proposal.

The discovery that OxsB and AlsB both make **2** [67], either as the product or as an intermediate has allowed us to hypothesize about the mechanism and reconsider the function of Cbl in these reactions. Although methylation using Me-Cbl has been reported for OxsB and AlsB [35,76], no methyl groups are found in **2**, **4** or **6**, suggesting that this methylation activity is an artifact. Additionally, all AdoMet consumption results in stoichiometric 5′-dA production with no SAH detected [67]. Importantly, the recent report by Lee, Fan, & Yeh et al. (2025) provide evidence that both OxsB and AlsB require two AdoMet molecules to generate two 5′-dA• species to perform two hydrogen atom abstractions in generating **2**. With the role of the RS component of OxsB and AlsB established, the role of Cbl can be reexamined. Previously, based on the OxsB structure, two possible roles for Cbl were proposed: lowering the activation energy barrier for the chemically challenging ring contraction step via Cbl-coordination of a substrate-bound radical species, or use of Cbl as an electron acceptor [31]. The latter seems like a poor use of an expensive cofactor. Now with the evidence that OxsB and AlsB generate **4** and **6** respectively using two rounds of RS chemistry, the covalent adduct possibility is even more attractive. Also, there is no longer a need for an electron acceptor (Fig. 7D).

In Lee, Fan, & Yeh et al. (2025), the authors propose a mechanism involving a substrate-cob(III)alamin adduct, where production of 5′-dA• takes place before and after adduct formation, with adduct formation allowing the enzyme to hold on to the reaction intermediate while the enzyme exchanges 5′-dA and methionine for a second AdoMet (Fig. 7D) [67]. Leveraging a nearby metallocofactor to stabilize a reaction intermediate amid a second enzyme turnover has previously been observed in tetraether lipid synthase [77] and in lipoyl synthase [78]. For OxsB, a C2′′ adduct with Cbl was proposed [67], however, the order of hydrogen atom abstraction is not known and a 4′ adduct with Cbl may form (Fig. 7D). Following the second hydrogen atom abstraction, the formation of a C4′-C2′ bond would break the attachment to the Cbl (Fig. 7D). In this proposal, Cbl may play more than one role. In addition to holding onto the reaction intermediate between half reactions via adduct formation, the Cbl could also facilitate the rearrangement of the ribose ring into the strained [2.1.0]-fused bicyclic compound, which is not straightforward chemistry. A substrate-cob(III)alamin adduct has been proposed previously in Cbl-dependent enzyme QueG, but isolation and characterization of the adduct has been untenable [49]. A substrate-cob(III)alamin adduct in OxsB has been proposed based on density functional theory calculations, though this proposal involves an O-cob(III)alamin adduct rather than a C-cob(III)alamin adduct [79]. The dehalogenase PceA has also been proposed to form a substrate-Co adduct after elimination of a chlorine anion from the substrate [47,80–83]. Cbl adducts are not a novel concept; adduct formation by free Cbl has been observed [84,85]. However, the involvement of Cbl in adduct formation as part of the catalytic mechanism of Cbl-dependent RS or non-RS enzymes is not yet fully vetted and awaits additional structural and/or spectroscopic evidence.

## 8. Conclusions

The research of the Cbl-dependent RS family has been full of exciting progress and surprising turns. In only eight years, the field has gone from the first solved crystal structure to engineering these enzymes to catalyze novel reactions [31,36,37]. Structures are now available with substrates bound (Table 1) showing large variations in the protein environment surrounding the Cbl [26,50,51]. Additionally, the recent discovery of a smaller 4 β-strand Cbl-binding domain in this family indicates both a substantial increased number of potential Cbl-dependent RS enzymes, and also a connection to the CFeSP of the Wood-Ljungdahl pathway, linking two separate branches of inorganic biochemistry [26,27,57]. Both the CFeSP and the class 2 Cbl-dependent RS enzymes use an uncharged or hydrophobic residue to occupy the space where we would expect a lower axial ligand [26,27,57]. As seen in the first structure of MetH in 1994, the Base-off Cbl-binding mode allows the protein to modulate the local environment of the Co [41], and now we are observing nature leverage that ability with a growing number of amino acids and environments [26,27,31,50,51].

Although OxsB was the first Cbl-dependent RS enzyme to be solved, a substrate-bound structure has yet to be visualized (Table 1). The lack of a substrate bound in the OxsB structure is likely due to the C-terminal domain being forced into an open conformation by lattice contacts [31]. A substrate bound OxsB structure or AlsB structure may finally answer the longstanding questions regarding these two enzymes. Specifically, a substrate or product bound structure could be the smoking gun for establishing the role of Cbl in OxsB, which has not been fully elucidated [35,67,76,79]. With the [2.1.0]-fused bicyclic compound characterized, new mechanistic proposals for AlsA can also be considered. It still remains to be determined how AlsA uses RS chemistry to remove a one-carbon unit.

Though the structural and biochemical work on Cbl-dependent RS enzymes has yielded incredible advances already, there are still many questions remaining. For example, what are the reactions catalyzed by DUF512 enzymes? Will we start to be able to predict the type of Cbl reaction from the identity of the lower ligand? Why do MetH and CFeSP use different Cbl-binding folds when their reactions are so similar? Will Cbl-dependent RS enzymes be discovered that use corrinoids outside of the Cbl subfamily? Will there be a common function for Cbl in non-methyltransferases? We remain in the early days in this field, with hard-earned structural and biochemical data pushing us forward, answering some questions and raising more questions. Cbl-dependent RS enzymes have been, and will likely remain, a treasure chest full of beautiful inorganic biochemistry with more surprises to come.

## Figures and Tables

**Table 1:** Current structures of Cbl-dependent RS enzymes and their observed active site constituents.

Enzyme	Active Site Constituents	PDB Accession codes
OxsB	Apo	5UL2
	Cbl, [4Fe-4S] cluster, Dithiothreitol	5UL3
	Cbl, [4Fe-4S] cluster, AdoMet	5UL4
TsrM	Cbl, [4Fe-4S] cluster	6WTE
	Cbl, [4Fe-4S] cluster, Aza-AdoMet, Substrate	6WTF
TokK	Cbl, [4Fe-4S] cluster, 5′-dA, Met	7KDX
	Cbl, [4Fe-4S] cluster, 5′-dA, Met, Substrate	7KDY
Mmp10	Cbl, [4Fe-4S] cluster, AdoMet, Substrate	7QBS
	Cbl, [4Fe-4S] cluster, SAH*	
	Cbl, [4Fe-4S] cluster, SAH*	7QBT
	Cbl, [4Fe-4S] cluster, SAH	7QBU
		7QBV
*cs*DUF512	Cbl, [4Fe-4S] cluster, Aza-AdoMet	9CG1
*pf*DUF512	Cbl, [4Fe-4S] cluster, SAH, 5′-dA	9CG2

* Only the *S*-methyl-5′-thioadenosine moiety of *S*-adenosylhomocysteine (SAH) could be modelled. 5′-[*N*-[3*S*]-3-amino-carboxypropyl]-*N*-methylamino]-5′-deoxyadenosine (Aza-AdoMet) and SAH were used as structural analogues of AdoMet.

## Abbreviations:

Cbl: Cobalamin
AdoMet: *S*-adenosylmethionine
RS: Radical *S*-adenosylmethionine
TIM: Triose phosphate-isomerase
5′-dA•: 5′-deoxyadenosine radical
MeCbl: Methylcobalamin
AdoCbl: 5′-deoxyadenosylcobalamin
DMB: 5,6-dimethylbenzimidazole
5′-dA: 5′-deoxyadenosine
SAH: *S*-adenosylhomocysteine
MetH: Methionine synthase
DUF: Domain of unknown function
PDZ: PSD-95, DLG and ZO-1
OHCbl: Hydroxocobalamin
CFeSP: Corrinoid iron-sulfur protein
ACS: Acetyl-CoA synthase
OMe-DMB: 5-methoxybenzimidazole
OXT-A: Oxetanocin-A
2′-dAMP: Deoxyadenosine-5′-monophosphate
2′-dADP: Deoxyadenosine-5′-diphosphate
2′-dATP: Deoxyadenosine-5′-triphosphate
HD: His-Asp
